# Disempowering women—a mixed methods study exploring informational support about pain persisting after childbirth and its consequences

**DOI:** 10.1186/s12884-022-04841-6

**Published:** 2022-06-23

**Authors:** Beata Molin, Sofia Zwedberg, Anna-Karin Berger, Anna Sand, Susanne Georgsson

**Affiliations:** 1 Department of Clinical Science Intervention and Technology (CLINTEC), Division of Obstetrics and Gynaecology, Karolinska Institutet, Karolinska University Hospital, 141 86 Stockholm, Huddinge Sweden; 2 Department of Health Promoting Science, Sophiahemmet University, Stockholm, Sweden; 3 Theme Children’s & Women’s Health, PA Pregnancy Care and Delivery, Karolinska University Hospital, Stockholm, Sweden; 4 The Swedish Red Cross University, Stockholm, Sweden; 5 Department of Women’s and Children’s Health, Karolinska Institutet, Stockholm, Sweden

**Keywords:** Childbirth, Chronic pain, Information, Empowerment, Mixed methods

## Abstract

**Background:**

Access to information is essential to achieving individual empowerment; meaning the ability to exercise control, manage one’s own condition and make informed decisions. However, studies have shown that information provided to women regarding physiological changes during the postpartum period and postpartum health was inadequate, incorrect, or inconsistent.

**Methods:**

The aim of this study was to explore informational support about pain persisting after childbirth and its consequences. A sequential explanatory mixed methods design was used. In the first, quantitative phase, 1,171 women, who gave birth eight months earlier, completed a self-administered questionnaire. In the second, qualitative phase, 20 women who experienced chronic pain were interviewed. Descriptive statistics and qualitative content analysis were used to analyse the data.

**Results:**

The majority of the women did not receive information about pain persisting after childbirth, or the information was insufficient or incorrect. They did not know when and where to seek help and did not consult health care professionals. In addition, the lack of information had a negative impact on women’s psychological well-being. All women expressed the need to be informed by health care professionals, irrespective of the individual risk of developing chronic pain.

**Conclusions:**

Health services should ensure availability of information to give the women opportunity to achieve empowerment to make good health decisions, increase control over their health and well-being as well as to enhance their self-efficacy. We propose that a booklet or leaflet with relevant information about the risk of developing chronic pain, symptoms and treatment, along with advice about appropriate health care settings should be provided as part of antenatal or postnatal care.

## Introduction

In previous research, up to 35% of women reported pain related to pregnancy or birth, 6 months to 12 years after childbirth [1–5]. Pain may have many negative consequences on women’s physical and social activities, psychological well-being, intimate relationships, and ability to care for their children [6–9]. Studies have also shown that, in general, individuals with chronic pain (pain with duration > 3 months) [10] are often at risk of developing complications, including physical and psychological dysfunctions, such as depression and anxiety disorders, impaired cognitive, physical and sexual functions as well as ischaemic heart disease and cerebrovascular disease [11].

Chronic pain has the capacity to be more complex in its pathophysiology with time and thus potentially more difficult to treat [11]. In general, contact with health care and adequate treatment are crucial to prevent development of chronic pain and long-term consequences [11, 12]. However, previous research has shown that individuals with chronic pain do not know where to seek professional support to manage their pain [13]. In addition, studies have reported that women often do not seek professional help for post-childbirth morbidities [5, 14, 15]. Barriers to women seeking support include lack of knowledge about the condition, being unaware of available treatment as well as stigma associated with the morbidity [14, 16–19].

Knowledge and information are fundamental to the process of empowerment and play an active role in improving health [20, 21]. WHO defines empowerment as “a process through which people gain greater control over decisions and actions affecting their health, and as such individuals and communities need to develop skills, have access to information and resources, and the opportunity to participate in and influence the factors that affect their health and well-being” [21]. Empowerment is a key element for improving health outcomes and high satisfaction with health care [22, 23]. Access to the right information, at the right time, delivered in the right way, plays a crucial role in a person’s active decision-making [20, 24]. If individuals have the capacity to make good health decisions, their ability to protect, maintain, and increase control over their health as well as their self-efficacy and ability to achieve change over their condition, are increased [23, 25]. However, studies have shown that the information provided to women regarding physiological changes during the postpartum period, postpartum health and well-being was inadequate, incorrect, or inconsistent, leading to complications and postponed recovery, which had a negative impact on the transition to motherhood [26–29]. Previous research, investigating women’s experiences of living with enduring perineal trauma, has reported that they did not know what symptoms are considered normal during the postnatal period [16, 18, 19, 30, 31]. Women who experienced persisting pregnancy-related pelvic girdle pain described it as unexpected [6, 8].

To our knowledge, this is the first study focusing on women’s perceptions and experiences of information regarding pain persisting after childbirth. By examining the informational support from the women’s perspective, we intend to gain a better understanding of their informational needs and identify gaps within maternal care. Hence, the aims of this mixed method study were: (1)to describe to what extent and which format women reported that they have received information regarding the risk of developing chronic pain related to childbirth from healthcare providers; (2) to investigate women’s experiences of the received, or lack of, information and its consequences; (3) to describe to what extent and which format women would like to receive information.

## Methods

### Study design and procedure

**Fig. 1:**
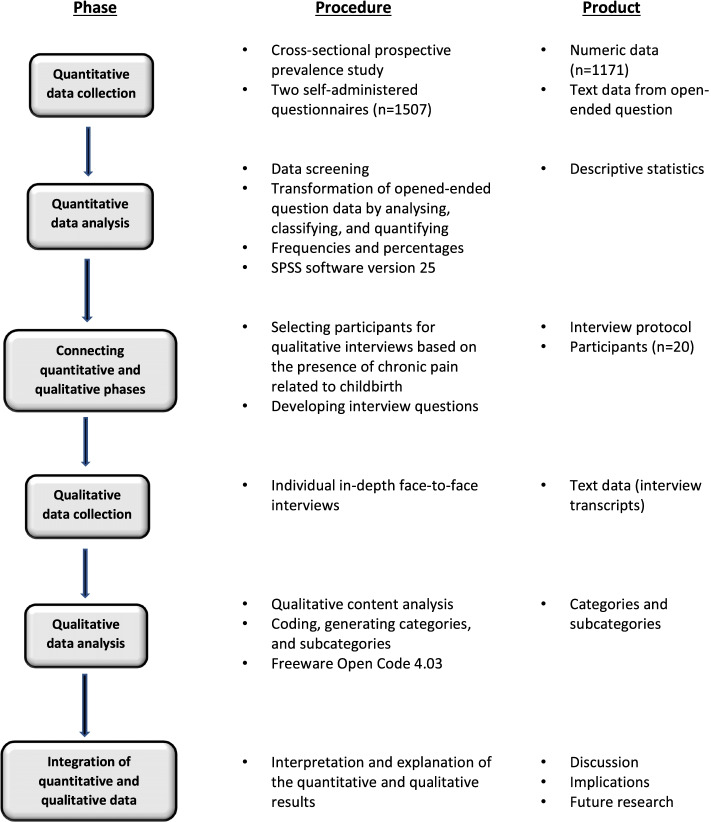
Model of the sequential explanatory mixed method study design

The mixed methods design was used to obtain both general and in-depth knowledge, and provide a more comprehensive understanding of the phenomena studied [32]. We used a sequential explanatory mixed methods design to confirm as well as further explore the results from a quantitative phase with insights gained from qualitative interview findings. This design entails collecting and analysing quantitative, and then qualitative data, in two consecutive phases within one study [32]. The first, quantitative phase, provided knowledge of to what extent, and in which format women received information about risk of developing chronic pain from healthcare professionals, as well as how many women sought healthcare due to the persisting pain. In addition, the quantitative phase provided the selection of participants for the qualitative follow-up interviews. The second, qualitative phase, contributed to a deeper understanding of the informational support and its consequences, as well as women’s preferences regarding the information. A nested sequential sampling was applied, as the individuals who participated in the qualitative sample were a subset of those who participated in the quantitative sample. The quantitative and qualitative phases were connected while selecting the participants for qualitative interviews as well as during the discussion of the results [32]. Priority was given to the qualitative phase of the data collection and analysis, despite it being the second phase [33]. Priority means “which approach, quantitative or qualitative (or both), a researcher gives more weight or attention throughout the data collection and analysis process in the study” [33]. Although the quantitative data collection was robust, the data were limited to a few questions, and the data analysis employed only one statistical technique, descriptive statistics. The goal of the qualitative phase was a deeper understanding and interpretation of the statistical results obtained in the quantitative phase. The mixed methods sequential explanatory design procedures are presented in Fig. 1.

### Ethical considerations

This study was carried out in line with the guidelines contained in the Helsinki Declaration [34]. Ethical approval was obtained from the Regional Ethics Review Board in Stockholm (Dnr 2015 / 236–31). The women obtained both verbal and written information about the study. They were informed that participation was voluntary and were assured that data would be treated confidentially. If the woman consented to participate, a written informed consent form was signed regarding both the quantitative and qualitative data collection followed by a second verbal consent prior to the interviews.

### Quantitative phase—questionnaire survey

#### Study design and setting

The quantitative phase was conducted as a cross-sectional study and is a part of a prospective, multicentre prevalence study that included 1,507 women recruited on maternity wards at seven hospitals located in Stockholm, the capital of Sweden, and in two medium-sized cities [3]. All women who did speak and read Swedish were invited to participate. The women completed two self-administered questionnaires, the first during 24–36 h after birth and the second eight months after childbirth. In the present study, we have included a convenience sample of 1,171 women who completed the second questionnaire. For full details, see Molin et al. [3].

### Sample size

We conducted a sample size calculation to analyse risk factors regarding pain following childbirth. To detect differences regarding risk factors of at least 10% between women with, or without, chronic pain with a statistical power of 80%, and based on a significance level of 5%, the target sample size was 1,000 women. The sample size ratio between the no pain group and the pain group was estimated to be nine with the support of existing studies [35, 36]. Assuming a 30% dropout, the sample size was determined to be 1,500 women.

### Data collection

Data were obtained through a self-administered questionnaire between December 2015 and June 2016. The questionnaire was developed by the research team, consisting of pain researchers, midwives, and an obstetrician, after a systematic literature review and validated through one-to-one interviews with 15 women. The questionnaires consisted of questions about demographic and social characteristics, baseline measures of common maternal morbidities, pain presence and its onset, as well pain intensity, frequency, bodily localization, and pain interference with activities of women’s daily life. In general, all items were well perceived and easily understood. None of the questions regarding informational support had to be modified or reworded. Women were asked if they had received any information about the risk of developing chronic pain after childbirth (yes/no), if so, how (verbally, in writing or both), by whom (a midwife, an obstetrician, both or other) and when this information was provided (at a maternity care unit during pregnancy checks, at maternity ward during the immediate postnatal period or at a maternity care unit after childbirth). Women were also asked if they had sought healthcare due to persisting pain (yes/no).

### Quantifying of open-ended question

The questionnaire contained the following open-ended question: “If you would like to receive information about the risk for development of chronic pain related to childbirth, how would you like to receive it?” The qualitative data containing the free text answers were transformed into quantitative data by analysing, classifying, and quantifying [37] allowing the creation of an additional variable, “Do the women wish to receive information about the risk for developing chronic pain related to childbirth?”. The main steps of the transformation included analysis of the messages in the free text answers, using content analysis and independent coding (classification) of the data into five categories by two researchers (BM and A-KB): 1. Yes, I would like to receive information, 2. No, I would not like to receive any information, 3. I don’t know, 4. I am happy with the information I have received and, 5. Answer is missing/no opinion. If the women described when and/or how they would like to receive the information (for instance verbally, in writing, by the midwife, the doctor, during pregnancy, during postpartum check-up) we coded the answer as 1. Once the data were qualitatively analysed and classified, they were then quantified using descriptive statistics.

### Data analysis

Descriptive statistics of the collected data are presented as numbers (frequencies) and percentages. Missing data were left out of the analyses and no imputation was performed. Therefore, the numbers of responders included in the analyses vary. Data were processed and analysed using the statistical program SPSS IBM (Statistical Package for the Social Sciences) version 25.

### Qualitative phase—In-depth interviews

#### Study design and sample

The qualitative phase was a part of a data collection investigating women’s experiences of chronic pain as well as of caregiver’s support and attitudes [7]. Data were collected through in-depth interviews and analysed using inductive qualitative content analysis [38].

**Fig. 2:**
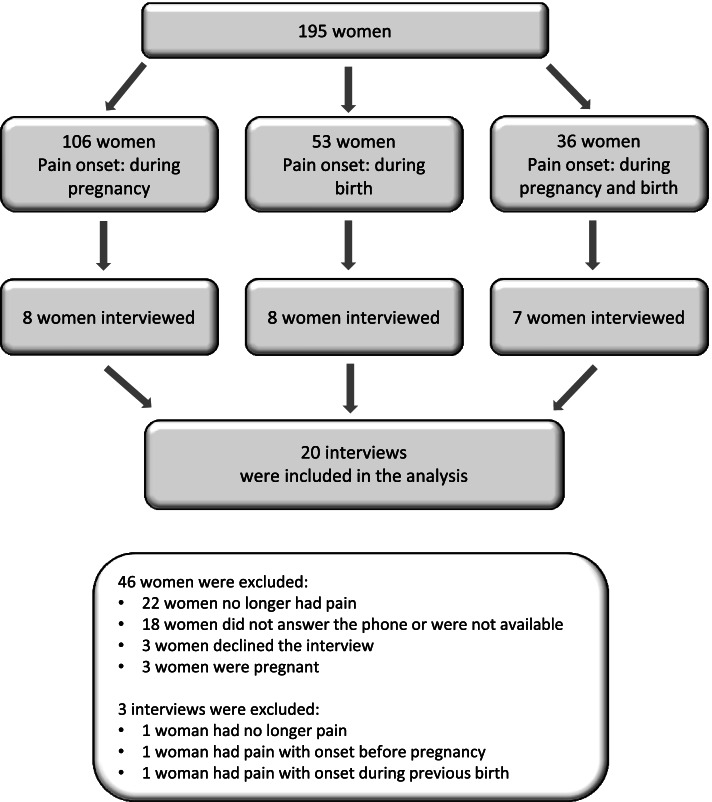
An overview of the selection procedure

The participants were selected from a subset of women from the quantitative data collection and included those who reported chronic pain eight months after childbirth. Chronic pain after childbirth was defined as any pain with onset during pregnancy or birth and still experienced at the time of the study. The women were divided into three groups, depending upon pain onset, and a random sample was selected from each group to obtain as much variety as possible in the material. An overview of the selection procedure is presented in Fig. 2. The sample size of 20 participants was considered sufficient to reach saturation [39]. By the seventeenth interview, the patterns in the women’s experiences were recognised and the final three interviews confirmed the sense that saturation had been reached. Three of the conducted interviews were not considered relevant to the study, hence, a further three women were selected according to the selection procedure and a total of 20 interviews were included in the analysis (see Fig. 2).

### Data collection

The women were interviewed in a single, face-to-face interview by the first author (BM) between June and November 2016. The main question in the interview guide was: “Can you tell me about your experience and thoughts regarding information about chronic pain?” Support questions (prompts) were used throughout, as needed, to ensure the depth and detail of the woman's story [40]. The interviews lasted between 15 and 56 min.

### Data analysis

The data were audio-recorded, transcribed verbatim, and analysed using qualitative content analysis. The focus of the analysis was to describe and present variations in the material with regard to similarities and differences [38]. The analysis was performed in a stepwise manner using the freeware Open Code 4.03 [15]. Each interview was read several times to obtain a sense of the whole picture, and the meaning units relevant to information about chronic pain after childbirth were identified. These units were condensed and coded to identify the manifest content of the text. Next, codes that shared a commonality were grouped into categories. Further, the categories were compared for similarities and differences and either divided into subcategories or merged into a new category. The first author was responsible for the analysis and the results were discussed among all the authors until a consensus of understanding of the data was reached.

## Results

### Questionnaire survey

In total, 1,171 (77.7%) out of 1,507 women responded to the second questionnaire and were included in the analysis. Table 1 shows the characteristics of the participants. Approximately 83% (954/1,155) reported that they had not received information about the risk of developing chronic pain related to childbirth. Of the 17% (201/1,155) who reported that they had received information, 84.8% (162/191) were informed by a midwife, 2.6% (5/191) by an obstetrician and 12.6% (24/191) received information from both. Furthermore, in 71.5% of cases (138/193), the information was given verbally, 9.8% (19/193) received written information, 17.1% (33/193) were informed both verbally and in writing, and 1.6% (3/193) did not remember. Of those women who experienced chronic pain related to pregnancy and/or birth (*n* = 195), 52.1% (100/192) did not seek health care for their problems. The analysis of the open-ended question showed that 81.7% of the women (957/1,171) would have liked to receive information about the risk of developing chronic pain after childbirth.

**Table 1:** Characteristics of the participants in the questionnaire survey (*n* = 1171)

	**n (%)**
**Country of birth** (*n* = 1,163)	
Sweden	1,013 (87.1)
Another European country	72 (6.2)
Country outside Europe	78 (6.7)
**Relationship status** (*n* = 1,162)	
Married or cohabiting	1,147 (98.7)
Single	15 (1.3)
**Occupational status** (*n* = 1,168)	
Employed	1,048 (89.7)
Student	46 (3.9)
Jobseeker	18 (1.5)
Other	56 (4.8)
**Education level** (*n* = 1,160)	
Elementary school	18 (1.6)
Upper secondary school	250 (21.6)
College	882 (76.0)
Other	10 (0.8)
**Parity** (*n* = 1,171)	
Primipara	685 (58.5)
Multipara	486 (41.5)
**Mode of birth** (*n* = 1,171)	
Vaginal	916 (78.2)
Instrumental	75 (6.4)
Spontaneous	841 (71.8)
Caesarean section	255 (21.8)
Emergency	119 (10.2)
Planned	136 (11.6)
**Perineal trauma**^a^ (*n* = 916)	
Degree 1 and 2	738 (80.6)
Degree 3 and 4	50 (85.4)
Episiotomy	7 (0.8)
	**Mean ± SD**
**Age** (year) (*n* = 1,171)	32.4 ± 4.5

^a^Of those women that had a vaginal birth (*n* = 916)

### In-depth interviews

Characteristics of the participants in the in-depth interviews are presented in Table 2. For confidentiality purposes the women have been given anonymous names. Three categories emerged in the interview data: 1. “Inadequate information “, 2. “Negative consequences”, and 3. “Information needs and requirements”.

**Table 2:** Characteristics of the participants in the in-depth interviews (*n* = 20)

*Pseudonym*	*Age (years)*	*Months since birth*	*Pain onset*	*Mode of birth*	*Parity*	*Country of birth*	*Educational level*	*Marital status*
Anna	28	9	Pregnancy + birth	Vaginal birth	Primipara	Sweden	College	Married
Cecilia	34	11	Birth	Vaginal birth	Multipara	Sweden	College	Married
Pia	36	13	Birth	Vaginal birth	Primipara	Sweden	College	Married
Elin	43	12	Birth	Caesarean section	Primipara	Sweden	College	Married
Ida	32	12	Pregnancy	Caesarean section	Primipara	Sweden	College	Married
Irene	25	9	Birth	Vaginal birth	Primipara	Sweden	College	Married
Emelie	36	10	Birth	Caesarean section	Primipara	Sweden	College	Cohabitant
Linda	39	11	Pregnancy + birth	Caesarean section	Multipara	Poland	Upper secondary school	Married
Rita	32	11	Birth	Vaginal birth	Multipara	Sweden	Upper secondary school	Married
Maria	40	9	Birth	Caesarean section	Primipara	Sweden	College	Married
Ester	41	11	Pregnancy + birth	Vaginal birth	Multipara	Sweden	College	Married
Mia	27	13	Pregnancy + birth	Vaginal birth	Primipara	Sweden	Upper secondary school	Married
Karin	36	11	Pregnancy	Caesarean section	Primipara	Mexico	College	Married
Frida	33	11	Pregnancy + birth	Caesarean section	Primipara	Chile	College	Single
Hanna	31	12	Pregnancy	Vaginal birth	Primipara	Sweden	Upper secondary school	Married
Laura	38	14	Pregnancy + birth	Vaginal birth	Multipara	Sweden	College	Married
Erika	31	14	Birth	Vaginal birth	Primipara	Sweden	College	Married
Paula	28	12	Pregnancy + birth	Vaginal birth	Primipara	Sweden	Elementary school	Married
Sara	35	14	Pregnancy	Vaginal birth	Primipara	Sweden	College	Married
Klara	27	13	Pregnancy	Vaginal birth	Multipara	Sweden	College	Married

### Inadequate information

#### Insufficient and incorrect information

Throughout the women’s stories, there was a recurring depiction that the women had not been provided with any information about the risk of developing chronic pain after childbirth. Most of the women who experienced pain during pregnancy were convinced that it would disappear after the baby was born. Those who had pain in the immediate postpartum period thought that it was natural to have pain for a short period after childbirth, but that it would then resolve.

I thought that it [pain] would disappear after I gave birth to my son, but it did not. Then I thought it might go away when everything has healed and contracted, but it has not done that either. (Paula)

Furthermore, the women described that they had not received any information about when they should seek help or how to manage their pain. In addition, they were not informed about appropriate health care providers to help if the pain persisted after childbirth.

No one said like, yes, if there is a problem with the scar, call this number and do this or think about it. I could not sit, I could not walk, I could not sleep because I was in pain—no one said anything about where I should turn. (Frida)

Several women felt that the information provided by health care professionals was inadequate in addressing their needs. For example, the women received information about certain risks, such as the consequences of sphincter ruptures or nerve damage during caesarean sections, but not about other conditions that had subsequently affected them. Furthermore, many of the women had been told that "it is normal to have pain after childbirth" but at the same time no one talked about what "normal" means and when the "normal” should no longer be considered "normal".

Then you do not know if it is normal, if it is ok, if it should be like this or not. After all, no one tells you what is normal or not. (Rita)

When the women asked health care providers for a more precise time indication regarding how long the pain following childbirth could persist, they were often told that it usually disappears after a few months. The women had also been told that the pain would resolve spontaneously and that they just had to “wait and see". In hindsight, the women experienced this information as simply incorrect because several of them still experienced pain after almost a year or more.

Now I'm sitting here a year later and I´m still in pain, and of course you wonder when it will disappear. No one has mentioned anything about how long I can have it, there does not seem to be anyone who knows. (Paula)

According to the women’s descriptions, information regarding the prevention, or treatment, of pain was general and not individualized. For example, they were informed that exercising is beneficial, but some experienced that physical training caused or worsened their pain. One woman reported that a brisk walk led to her pelvic joints locking so that she could barely move, resulting in a lot of pain. However, she continued to exercise to an even greater extent because she was told that it was beneficial—which made the situation even worse. Almost all women were informed that it is good to perform pelvic-floor exercises to prevent or treat pain, but the exercises had no effect in most cases.

At the hospital, they mainly talk about pelvic-floor exercises. "Just remember to work your pelvic floor" was the last thing they said before I went home. And then at the check-up the midwife said, "keep on working on your pelvic floor". That was basically it. I felt that the midwife was very limited in what she knew about pain. It was "working on pelvic floor is good to do" before, during and after pregnancy. (Ida)

#### Seeking other sources

Many women sought information from other sources. They often asked acquaintances and friends for advice or sought information on the internet, for example, on social media. Several women found groups on Facebook where they met women with similar problems. In these groups, the women often received acknowledgement, which made them feel less isolated and alone. The group members also provided the women with helpful information, for example, about where and how they could get help from health care professionals as well as about self-care management of pain. For several women, the internet was the first source of information to confirm that the persisting pain is not a normal condition and that they should seek help.

Unfortunately, I have let it be and thought that it will disappear sometime. But I have started to read a bit and it says that you should not go that long when you are in pain, so I will try to get to grips with it. (Hanna)

### Negative consequences

#### Emotional distress

Lack of, inadequate or incorrect information about pain persisting after childbirth led to emotional distress and a negative impact on the women's psychological well-being. Most expressed that the persistent pain was unexpected and that they felt unprepared and overwhelmed by it. This often led to feelings of despair, frustration, and anger. Some of the women were convinced that the pain condition they suffered from was very rare and many times they struggled with feelings of loneliness and isolation. Furthermore, several felt disappointed with, or even deceived by, the information from health care providers because their pain did not resolve and was still present one year after childbirth. One woman reported that she started to doubt her own experiences to the degree that she thought that the pain was in her imagination.

It is almost as if you think you are imagining it. It feels like I obviously did something wrong because I—even though everything went so well—am in so much pain. (Mia)

Some of the women were convinced that it was their own fault that they were still in pain and felt like a failure. They also blamed themselves because they should have understood that something was wrong and that the pain was not a normal condition. One woman expressed that she felt naive and stupid because she had believed that the pain would disappear on its own:

It's a bit my fault, I should have understood in some way that it is not just the body that fixes everything magically. I feel a little like a failure. Stupid and blue-eyed because I thought so. (Selma).

The fact that the women did not know if something was wrong with them, what was wrong or how the pain would progress over time, could lead to anxiety. They were afraid that the pain would worsen over time and that they may never be pain-free again. Several said that information would have reduced their anxiety and thereby their suffering. One woman pointed out that all the uncertainty led to high levels of stress, which in turn led to increased pain, like a vicious circle.

There was too little information on all levels. Then you just get even more stressed, and that does not mean that you feel less pain, of course. (Emelie)

#### Postponed seeking care

Lack of awareness about the risk of developing chronic pain related to pregnancy or birth, or belief that persisting pain is a natural consequence of childbirth, led to the women not recognising it as a condition that required medical attention and thus not seeking healthcare. Instead, the women thought that pain was simply something they had to endure, and they just waited for the pain to resolve spontaneously. Some of the women tried to ignore or deny their pain until finally it was unbearable.

I went into a bit of denial at the same time as I was in pain all the time, a bit like "yes, yes, it will surely go over". And then it just got worse and worse instead. It was quite difficult in the end. I was almost ready to cry before I decided to seek help. (Cecilia)

In general, women were confused about which health care settings were available and appropriate. They felt that the maternity clinic was not the right place after the pregnancy, the child health care centre focuses on the child, and the general practitioners in primary care do not have competence in gynaecology. This led to the women feeling excluded from the healthcare system. Some women said that the fact that they did not know where to seek help led to delays in getting appropriate and timely treatment.

I was really scared that they had missed something. And it's so hard to find people to help too and to know where to turn. If I knew, I think I could have recovered much earlier. (Pia)

When some of the women finally sought medical care, they did not receive adequate support or further information. Therefore, after trying, they gave up further attempts.

I got an oestrogen ointment, so I used it, but I do not know if it made much difference to be completely honest. And then I just waited and waited and thought, but maybe it's getting better, maybe it's getting better, like that. But all the time I thought I may have to find out where to go, where to turn if there is anything wrong with me. I think I'm going to do something, but I do not really know what, so no, I have not done anything. (Ester)

To discuss urogenital problems openly was experienced by the women as taboo and stigmatizing. Some women explicitly mentioned embarrassment and shame as impediments which raised the threshold for consulting a health care professional.

It's a little embarrassing that it hurts when I defecate. It's not something you walk around with a sign about over your head. So, I have sort of hesitated to contact someone over it, in fact, which is a bit silly, because if you don’t ask for help when you have this kind of pain related to childbirth, it is not easier to get help because everyone walks around and is ashamed. (Mia)

### Information needs and requirements

Almost all women expressed the need and desire for more information about persistent pain after childbirth and emphasised the responsibility of health care professionals in providing it. The information should be given irrespective of the potential risk of developing chronic pain and include what symptoms the women should be aware of, when and where to seek help and how pain can be prevented and treated. The information should be clear and explicit. Furthermore, it should be given by a midwife or an obstetrician and provided to the woman and her partner. When it comes to when this information should be given, many agreed that it needs be given during pregnancy, as there is often more time to absorb information and because after the birth there is so much else to focus on. The women said that the information should be preferably written, as a brochure or book, which they could read when appropriate and which they could return to if necessary.

Something in the form of a slim book. In it could be a section at the end regarding pain after childbirth or something like that. Then I would have read it. (Cecilia)

## Discussion

Our main quantitative results showed that most of the women did not receive any information about the risk of developing chronic pain after pregnancy or birth, and that half of them did not turn to healthcare with their problems. The qualitative analysis confirmed the quantitative results and, in addition, showed that the women were told that pain is a natural consequence of childbirth, and would resolve spontaneously. Furthermore, the women did not receive information about where to seek help. The inadequate and incorrect information could also lead to emotional distress. It has also emerged that most of the women would have liked to receive information about pain persisting after childbirth.

Insufficient and incorrect information may lead to women not being aware that pain persisting after pregnancy or birth is a condition that requires medical attention. Our results are in accordance with previous studies reporting that women often do not seek professional help for post-childbirth morbidities because they think that their morbidity is normal, will resolve over time, and/or are unaware of available treatment [14, 16–18, 41, 42]. However, as we have shown, chronic pain related to pregnancy or birth is not uncommon as one in six women reported chronic pain eight months after childbirth, and one in five experienced dyspareunia [3]. Chronic pain develops because of changes in the central nervous system initiated by acute pain, such as increased communication between neurons, or dysfunction in the descending inhibitory system [11, 43, 44]. If the initially reversible changes in the nervous system are left untreated, they can become irreversible with time, and the pain will be ultimately refractory to treatment. Therefore, pain researchers and clinicians emphasize the importance of timely and appropriate treatment—not only to decrease the negative impact of pain on quality of life and to reduce suffering—but also to prevent the development of chronic pain and reduce its associated long-term sequelae [11, 12]. We believe that if women received appropriate information about pain persisting after childbirth, they would more often consult healthcare professionals. Therefore, the opportunity to diagnose and treat, as well as prevent the development of chronic pain would increase. This is in accordance with previous studies showing that delayed help-seeking behaviour leads to delayed diagnosis and treatment and, as a consequence, to poor health outcomes [45].

In the in-depth interviews, the women also expressed that lack of adequate and accurate information left them unprepared for the pain, which contributed to emotional distress as they felt overwhelmed, anxious, angry, and frustrated. This is in line with previous studies showing that women with enduring symptoms after pregnancy or perineal trauma, described that lack of information resulted in a negative impact on psychological well-being and that the emotional distress was exacerbated when they were unprepared [8, 19, 46]. Furthermore, some of the women in our study described that emotional distress could also worsen their pain. This is consistent with well-established knowledge, that pain is a multidimensional phenomenon, and that the affective and cognitive aspects play a significant role in how individuals experience it [44, 47, 48]. In general, perception of pain may be enhanced by negative emotions and diminished by positive feelings [47, 49]. In addition, the experience of pain can be modified by expectations, and when the women’s expectations are not met, it may enhance their symptoms [8, 47]. Negative emotions can also impact individuals’ ability to cope with pain as they reduce opportunities to control it. Studies have shown that patients who believe they can control their pain are less likely to report that it interfered with daily functioning and appear to adjust to the pain better than those who do not [50]. This is in line with Wuytack et al. [8], who reported that it would be easier for women with persisting pregnancy-related pelvic girdle pain to cope with and manage their pain if they have been given more information about the enduring symptoms.

Most of the women, more than 80% in the quantitative part, and all the women participating in the interviews, agreed that the risk of developing chronic pain should be discussed prior to, or after, childbirth. The information should be given by a midwife or an obstetrician preferably already during pregnancy, as there is often more time to absorb information. According to the quantitative data, the majority of the women receive the information orally but, as it has emerged from interviews, they would prefer to obtain written information. The women were very clear that the information should be given irrespective of the individual risk of developing chronic pain. This is in accordance with previous studies showing that most women need information and want to be prepared for what can happen to them during the postnatal period [8, 16, 29, 51]. As our results highlight, there is a gap between women’s need of information about pain persisting after childbirth and the information received. According to previous studies, health care professionals are more concerned about this need of information during prenatal, rather than postpartum, care as well as prioritising the infants’ needs and not those of mothers [8, 41, 51]. According to the Swedish Patient Act [52], all patients have the right to equal and respectful treatment, self-determination, and participation. This includes the right to be informed about their condition, care alternatives, and possible treatment, including risks of complications as well as methods for preventing illness or injury. The information should be individually adapted to the recipients, and as far as possible, healthcare providers should ensure that the patient has understood the contents and significance of the information [52]. Access to information is essential to achieving patients’ empowerment. [22, 53]. According to Cerezo et al. [22], empowerment may be seen as an “enabling process whereby health care professionals collaborate with patients to help them acquire knowledge and resources and whose outcome is a patient with greater ability to exercise control, manage his/her own condition and to make an informed decision”. Absence of empowerment leads, in turn, to powerlessness, helplessness, loss of autonomy as well as lower quality of life [23, 53, 54]. There is a consensus among experts that we cannot afford not to empower patients, especially those with chronic diseases, as improving their ability to understand and manage their own disease as well as navigate the health systems is crucial to achieving better health outcomes [55].

Our results suggest the need for health services to ensure availability of information about pain persisting after childbirth. The lack and inadequacy of the information must be corrected. The purpose of the information is not to frighten women but to alert them. We propose that a booklet or leaflet containing relevant information about the risk of developing chronic pain, symptoms, and treatment, along with advice about appropriate health care settings where women can turn to, should be available. This should be sent home with the woman as a part of antenatal or postnatal care, for instance, at the postpartum check-up. Furthermore, it is crucial to have a multidisciplinary approach in this matter, with midwives, obstetricians, physiotherapists, and psychologists involved, because of the complexity of the problem. It is time to respect women’s rights to be informed about pain persisting after childbirth and give them the opportunity to achieve empowerment, to make good health decisions, and increase control over their health and well-being as well as their self-efficacy.

### Methodological considerations

This study used mixed methods, where the quantitative part supported the selection of participants for the qualitative in-depth interviews. The qualitative phase supported the discussion of the outcomes of the entire study (i.e., the interpretation of the quantitative data/results and in-depth understanding of the qualitative research objectives). By using different types of data collection, a researcher can provide more evidence with which to answer research questions as well as enhance the ability to draw conclusions [32]. The merged results of this mixed method study showed that the findings from the qualitative phase agreed (convergence) with the quantitative part in that most of the women did not receive the information they wanted. Furthermore, the qualitative findings offered in-depth knowledge about women’s experiences of information. They enriched the quantitative results and provided information about the consequences of missing and incorrect information such as that that the lack of information could lead to psychological distress and postponement of care (complementarity) [56]. However, although we describe consequences as a result of inadequate and incorrect information, there may be other reasons for delayed care seeking. For instance, socioeconomic status depending on profession, education, and employment conditions may influence the women’s experiences. In previous studies, women described various practical barriers, related to financial and time constraints, that prevented them from getting professional help, including the cost of treatment and finding someone to care for the baby. It is therefore possible that being unemployed and having a lower household income can be associated with a higher risk of not receiving professional help [41]. These, and other socioeconomic factors, should be investigated further. Due to the inclusion criteria, only 13% of the women in the quantitative part, and three women in the qualitative part of the study were foreign-born with a non-Swedish speaking background. This constitutes approximately half of the total of foreign-born mothers in Sweden (27.5%) [57]. The recruitment of participants also resulted in an under-representation of women with a lower educational level. In previous research, language differences have been identified as a barrier to seeking help for maternal morbidities. Furthermore, women from diverse cultures and with low health literacy were less likely to seek professional help due to a lack of knowledge in recognising the problem or thinking that the morbidity was normal. In addition, high-level education was seen as an enabler empowering women to seek help [41]. Therefore, including more women from ethnic minorities in our study might have provided a different perspective and should be investigated in future studies. Furthermore, the women who decided to volunteer in the qualitative phase of the study may have been those who experienced the most extensive impact of pain on their lives. This limits the extent to which these findings can be generalized or transferred to other patients and settings or populations [32, 58]. Further, a self-developed survey instrument was used; although we validated and tested the questionnaire through one-to-one interviews with 15 women. Recall bias may also have influenced the results as the women gave birth to a child eight months earlier. However, it is known that women have a good recollection of their pregnancy, birth and received maternity care even years after the childbirth [59]. In qualitative research methods, reliability and validity are discussed in terms of trustworthiness, which includes confirmability, dependability, and credibility [58, 60]. To strive for confirmability, the process of analysing qualitative data has been described in detail with citations provided. The credibility of the data was established by various constellations of research group members holding discussions throughout the analysis process [60]. As the first author´s background is that of a midwife but also a pain educator, her preconceived knowledge could be disadvantageous during data collection and analyses. However, the researchers were aware of this situation and the involvement of co-authors counterbalanced this pre-understanding and contributed to a balanced discussion of the data [60].

## Conclusions

This study provides new knowledge about informational support regarding pain persisting after childbirth. The majority of the women did not receive information about pain persisting after childbirth, or the information was insufficient or incorrect. They did not know when and where to seek help and half of the women did not consult health care professionals. Furthermore, the lack of information had a negative impact on women’s psychological well-being. All women expressed the need of being informed by health care professionals irrespective of the individual risk of developing chronic pain. Health services should ensure availability of information to give the women an opportunity to achieve empowerment, to make good health decisions, increase control over their health and well-being as well as to enhance their self-efficacy. We propose that a booklet or leaflet with relevant information about the risk of developing persistent pain, symptoms, and treatment along with advice about appropriate health care settings should be provided as a part of antenatal or postnatal care. 

## Data Availability

The datasets generated and/or analysed during the current study are not publicly available since participants did not give consent for public sharing of their information and have been promised confidentiality but are available from the corresponding author on reasonable request. The data are protected by confidentiality in accordance with The Public Access to Information and Secrecy Act (SFS 2009:400) [61] and handled in accordance with the Personal Data Act (SFS 1998:204) [62].
